# Post-diagnostic ultra-processed food exposure in gastrointestinal cancers: scoping review with narrative synthesis and clinical implications

**DOI:** 10.3389/fnut.2026.1884359

**Published:** 2026-07-14

**Authors:** Ester Oneda, Silvia Noventa, Michela Libertini, Sara Cherri, Alessandra Manno, Fausto Meriggi, Fausto Petrelli, Alberto Zaniboni

**Affiliations:** 1 Oncology Department, Istituto Ospedaliero Fondazione Poliambulanza, Brescia, Italy; 2 Oncology Unit, ASST Bergamo Ovest, Treviglio, Italy

**Keywords:** cancer survivorship, colorectal cancer, gastrointestinal cancers, gut microbiome, nutritional epidemiology, ultra-processed foods

## Abstract

**Background:**

Modifiable lifestyle factors, including diet quality, are strongly associated with the incidence of gastrointestinal cancers. However, the impact of ultra-processed food (UPF) consumption after cancer diagnosis remains poorly understood, with scarce and fragmented evidence. In particular, the role of post-diagnostic UPF exposure in shaping survival outcomes and disease progression has not been systematically explored.

**Methods:**

We conducted a scoping review with narrative synthesis of the available literature on post-diagnostic UPF consumption and clinical outcomes in gastrointestinal cancers. Eligible studies included adult patients with gastrointestinal malignancies, assessment of dietary exposure after diagnosis, and outcomes such as overall survival, cancer-specific mortality, recurrence, progression, and treatment-related outcomes.

**Results:**

Direct evidence was extremely limited. The only available prospective study in colorectal cancer survivors showed that higher post-diagnostic UPF intake was not associated with overall or cancer-specific mortality but was associated with increased cardiovascular mortality, highlighting the relevance of competing risks in cancer survivorship. Furthermore, specific UPF subgroups showed adverse associations with colorectal cancer-specific mortality. Supportive studies suggested that dietary quality may deteriorate after treatment, with increasing UPF consumption over time. Mechanistic evidence supports a biologically plausible link between UPF exposure, metabolic dysfunction, chronic inflammation, microbiota alterations, and survivorship outcomes.

**Conclusions:**

Despite the increasing burden of gastrointestinal cancers and the widespread consumption of ultra-processed foods, post-diagnostic UPF exposure remains largely overlooked in oncologic research. Direct evidence is currently limited and mainly restricted to colorectal cancer survivors, where higher UPF intake has been associated with cardiovascular mortality but not consistently with cancer-specific outcomes. These findings, together with biological plausibility from mechanistic studies, support the need for prospective post-diagnostic cohorts and intervention trials integrating standardized dietary assessment into gastrointestinal cancer survivorship research.

## Graphical Abstract

**Figure:**
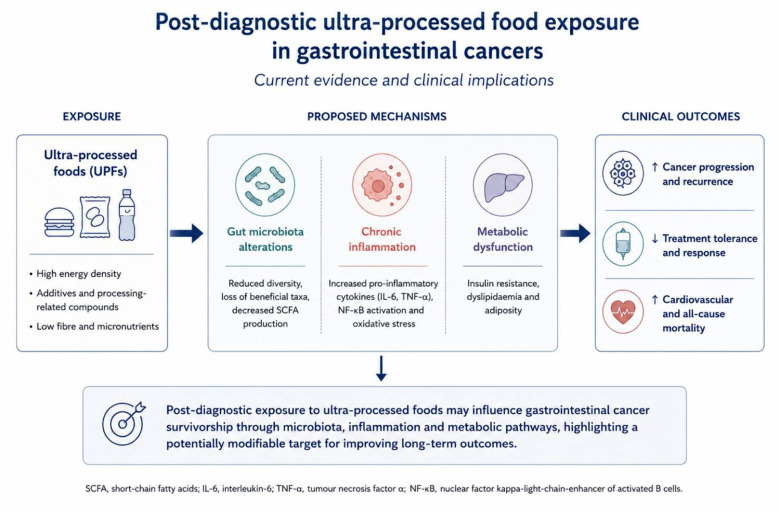

## Highlights

- Evidence on post-diagnostic UPFs in GI cancers remains limited.
- Higher UPF intake was linked to cardiovascular mortality in CRC survivors.
- Diet quality may worsen after gastrointestinal cancer treatment.
- UPFs may affect survivorship through metabolic and microbiota pathways.
- Nutritional strategies deserve investigation in GI survivorship care.

## Introduction

Gastrointestinal cancers remain a major cause of cancer-related mortality worldwide despite advances in systemic therapies and early detection strategies (1).

Alongside genetic and environmental determinants, growing evidence indicates that dietary habits substantially influence gastrointestinal carcinogenesis. In particular, Western dietary patterns and high consumption of ultra-processed foods (UPFs) have been consistently associated with increased risk of colorectal and other gastrointestinal cancers (2–7). According to the NOVA classification, UPFs are industrial formulations made mostly or entirely from substances extracted from foods, derived from food constituents, or synthesized in laboratories, with little or no intact whole food. They commonly contain additives such as emulsifiers, colorants, flavor enhancers, sweeteners, stabilizers, and preservatives, and include products such as sugar-sweetened beverages, packaged snacks, reconstituted meat products, instant foods, and many ready-to-eat meals. Importantly, UPF exposure should be distinguished from broader dietary constructs such as the Western dietary pattern or overall diet quality. While these constructs often overlap, NOVA classifies foods according to the extent, purpose and nature of industrial processing rather than nutrient composition alone.

The biological rationale underlying this association is increasingly supported by mechanistic and translational evidence. UPFs are typically characterized by high energy density, poor nutritional quality, low fiber content, and the presence of additives and processing-related compounds. These dietary patterns rich in UPFs are associated with chronic low-grade inflammation, metabolic dysfunction, insulin resistance, altered insulin/IGF-1 signaling, and gut microbiota dysbiosis, all of which may contribute to carcinogenesis and disease progression (1, 8–12). In particular, diet-induced alterations in gut microbiota composition and intestinal barrier integrity have emerged as potentially important mediators linking nutrition, systemic inflammation, and gastrointestinal cancer biology (13–16).

Beyond its established role in cancer prevention, diet has emerged as a potentially important determinant of survivorship. Dietary patterns may influence treatment tolerance, symptom burden, body composition, metabolic health, cardiovascular risk, and quality of life, all of which contribute to long-term outcomes in cancer survivors. While dietary interventions are increasingly incorporated into supportive care programs, the specific impact of post-diagnostic ultra-processed food exposure remains poorly characterized. However, growing interest in survivorship medicine and lifestyle interventions suggests that modifiable behavioral factors may continue to shape outcomes well beyond the initial diagnosis.

Therefore, this review provides a focused synthesis of the available evidence regarding post-diagnostic UPF exposure and gastrointestinal cancer outcomes, while discussing the potential role of inflammation, microbiota alterations, and metabolic dysfunction in survivorship and disease progression.

## Methods

### Study design and review framework

This study was conducted as a focused scoping review with systematic literature search and narrative synthesis. A scoping review design was selected because the available evidence on post-diagnostic ultra-processed food (UPF) exposure in gastrointestinal cancer populations is limited, heterogeneous, and unevenly distributed across cancer sites, exposure definitions, and clinical outcomes. The objective was therefore to map the extent, nature, and characteristics of the available evidence rather than to provide a quantitative estimate of effect or establish causal associations.

The review was informed by the methodological framework originally proposed by Arksey and O'Malley and subsequently refined by Levac et al. and the Joanna Briggs Institute guidance for scoping reviews. Reporting was guided by the Preferred Reporting Items for Systematic Reviews and Meta-Analyses extension for Scoping Reviews (PRISMA-ScR). A completed PRISMA-ScR checklist is provided as Supplementary Table 1S.

The research question was formulated according to the population–concept–context (PCC) framework: What is the extent, nature, and distribution of the available evidence on post-diagnostic UPF exposure in adults with gastrointestinal cancers during treatment and survivorship?

The PCC elements were defined as follows. The Population included adults diagnosed with gastrointestinal malignancies, including colorectal, gastric, oesophageal, pancreatic, liver, and biliary tract cancers. The Concept was post-diagnostic exposure to UPFs, defined as dietary exposure occurring after cancer diagnosis, during treatment, post-treatment follow-up, or survivorship. When available, UPF exposure was defined according to the NOVA classification, which categorizes foods according to the nature, extent, and purpose of industrial processing. Studies evaluating closely related but non-equivalent dietary exposures, such as post-treatment diet quality, Western dietary patterns, or dietary interventions not specifically classified by NOVA, were considered separately as indirect or contextual evidence. The Context included gastrointestinal oncology care, active treatment, post-treatment follow-up, and cancer survivorship.

### Search strategy and information sources

A structured literature search was conducted in PubMed/MEDLINE and supplemented by manual screening of reference lists from relevant articles, reviews, and meta-analyses. No additional bibliographic databases were searched. This approach was consistent with the focused scope of the review and the objective of mapping the available direct and supportive evidence rather than conducting an exhaustive systematic review.

The search period covered studies published from January 2009, corresponding to the broader implementation of the NOVA classification framework, through March 2026. Only English-language publications involving human subjects were considered.

The PubMed/MEDLINE search combined terms related to ultra-processed foods, gastrointestinal cancers, post-diagnostic dietary exposure, survivorship, prognosis, recurrence, mortality, and treatment outcomes. The search strategy used combinations of free-text terms and MeSH terms when available. The full PubMed/MEDLINE search strategy is reported in Supplementary Table S2.

Manual reference screening was performed by reviewing the reference lists of key eligible studies and relevant reviews to identify additional articles providing direct post-diagnostic evidence, indirect survivorship evidence, or contextual epidemiological and mechanistic evidence.

### Eligibility criteria

Eligibility criteria were defined before study selection. Studies were considered directly eligible if they included adult patients aged ≥18 years with gastrointestinal malignancies, assessed dietary exposure after cancer diagnosis, evaluated UPF consumption directly or according to NOVA-compatible principles, and reported clinical, prognostic, or survivorship-related outcomes. Eligible gastrointestinal malignancies included colorectal, gastric, oesophageal, pancreatic, liver, and biliary tract cancers. Observational and interventional human studies were considered.

Studies were excluded from the direct evidence category if they evaluated only cancer incidence without post-diagnostic assessment, assessed exclusively pre-diagnostic dietary exposure, involved pediatric populations, were exclusively preclinical, or were reviews, editorials, protocols, conference abstracts without sufficient data, or non-peer-reviewed reports.

Because direct post-diagnostic evidence was expected to be limited, additional sources were included as indirect or contextual evidence when they were relevant to the review question. These included studies in mixed cancer-survivor populations, post-treatment dietary-quality studies, dietary intervention studies not specifically designed to reduce UPF intake, incidence-based epidemiological studies, systematic reviews/meta-analyses, and mechanistic or translational studies. These sources were not treated as equivalent to direct post-diagnostic evidence but were used to contextualize biological plausibility, identify research gaps, and support interpretation.

For synthesis, included sources were classified into three evidence categories:

- direct post-diagnostic evidence in gastrointestinal cancer populations;
- indirect survivorship evidence from mixed cancer populations or broader dietary-quality studies;
- contextual epidemiological, mechanistic, and translational evidence.

### Study selection process

**Figure 1:**
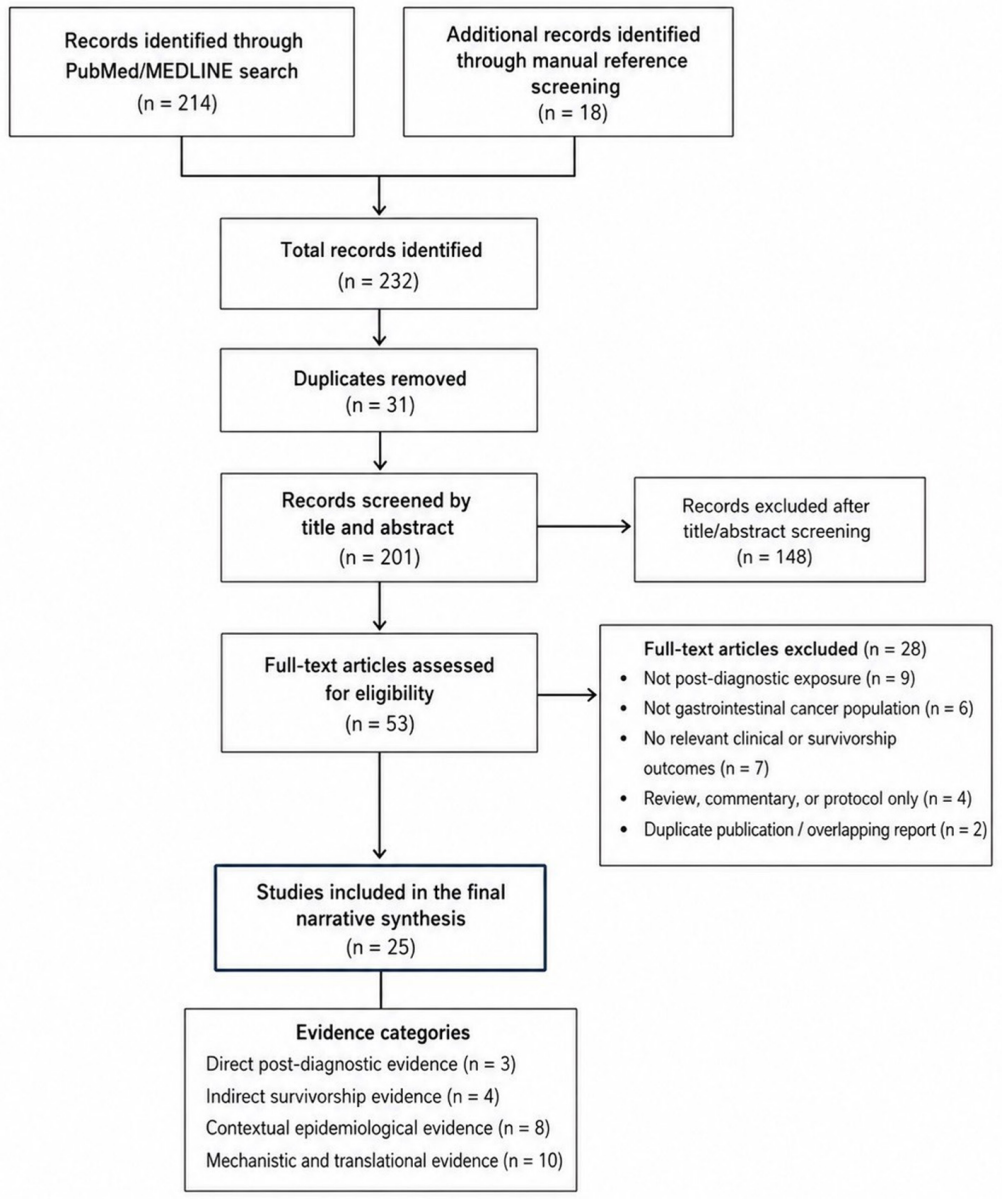
PRISMA flow diagram. PRISMA flow diagram showing the literature identification and selection process for studies included in the scoping review. Records were identified through PubMed/MEDLINE searching and manual reference screening.

The study selection process is shown in Figure 1.

### Data extraction

Data extraction was performed using a predefined standardized framework. The following information was collected when available: first author and publication year; country and study setting; study design; sample size; cancer type and stage; timing of dietary assessment; dietary assessment methodology; UPF classification approach; duration of follow-up; clinical or survivorship outcomes assessed; effect estimates and confidence intervals; and covariate adjustment.

For contextual epidemiological and mechanistic evidence, extracted information focused on study design, population, cancer site, dietary exposure, main findings, and relevance to post-diagnostic survivorship.

Given the heterogeneity of included sources, study designs, exposures, and outcomes, findings were synthesized narratively rather than quantitatively pooled.

### Methodological appraisal

Methodological limitations of included observational studies were assessed qualitatively, with attention to selection bias, dietary assessment methodology, exposure classification, confounding adjustment, outcome ascertainment, and relevance to the post-diagnostic setting.

Because the available evidence was predominantly observational and heterogeneous, and because the aim of this scoping review was to map the evidence rather than to estimate pooled effects, formal quantitative risk-of-bias scoring was not performed. The strength and relevance of evidence were instead interpreted according to study design and evidence category, with direct post-diagnostic studies considered separately from indirect survivorship and contextual evidence.

### Evidence hierarchy

For the purpose of synthesis, direct post-diagnostic studies in gastrointestinal cancer populations were considered the highest-relevance evidence category. Studies evaluating broader survivorship populations, post-treatment diet quality, dietary interventions, cancer incidence, or mechanistic pathways were used as indirect or contextual evidence and were interpreted separately. This hierarchy was applied to avoid treating epidemiological, mechanistic, and survivorship data as equivalent sources of evidence.

## Results

### Direct post-diagnostic evidence in gastrointestinal cancer populations

Direct post-diagnostic evidence was defined as studies assessing UPF exposure, or closely NOVA-compatible dietary exposure, after cancer diagnosis in patients with gastrointestinal malignancies and reporting clinical or survivorship-related outcomes. This evidence category was extremely limited and was almost entirely restricted to colorectal cancer survivors.

#### Colorectal cancer

Colorectal cancer is currently the only gastrointestinal malignancy for which direct post-diagnostic evidence is available. Among the identified studies, the prospective cohort by Hang et al. represents the most directly relevant investigation of post-diagnostic UPF consumption in gastrointestinal oncology. The study included 2,498 patients with stage I–III colorectal cancer, assessed diet at least 6 months after diagnosis using validated food-frequency questionnaires, applied the NOVA classification, and evaluated colorectal cancer-specific, cardiovascular, and overall mortality using multivariable-adjusted Cox models. Post-diagnostic UPF intake (6 months - 4 years after diagnosis) was not associated with colorectal cancer-specific mortality or all-cause mortality overall, but higher intake was associated with significantly increased cardiovascular mortality (1). The same study also reported heterogeneity across UPF subgroups: higher consumption of fats/condiments/sauces was associated with greater cardiovascular mortality, while higher intake of flavored yogurt/dairy-based desserts, particularly ice cream/sherbet, was associated with increased colorectal cancer-specific mortality (1). These findings suggest that total UPF burden may mask clinically relevant differences between specific UPF categories.

Supportive post-treatment evidence also comes from a prospective study in colorectal cancer patients after colostomy, which showed worsening diet quality over time, including reduced consumption of minimally processed foods and increased ultra-processed food intake during follow-up (17). Although this study did not assess survival or recurrence, it is highly relevant because it suggests that the post-treatment period may be characterized by dietary deterioration toward patterns plausibly associated with adverse long-term outcomes.

A feasibility randomized trial in bowel cancer survivors further supports the modifiability of this exposure. In HEAL ABC, a lifestyle intervention improved diet quality and reduced UPF consumption by about 10% of total energy intake, supporting the feasibility of targeting this dietary dimension in survivorship care, although the trial was not powered for recurrence or survival endpoints (18).

#### Other gastrointestinal cancers

For gastric, pancreatic, oesophageal, liver, and biliary tract cancers, no studies directly evaluating post-diagnostic UPF exposure in relation to recurrence, treatment response, survival, or survivorship outcomes were identified. Evidence for these cancer sites was therefore considered indirect or contextual and is summarized separately below.

### Indirect survivorship evidence

Indirect survivorship evidence included studies evaluating post-treatment diet quality, UPF-related dietary patterns, nutritional behaviors, symptoms, quality of life, or mortality in broader cancer-survivor populations. Although these studies do not provide direct evidence specific to post-diagnostic UPF exposure in gastrointestinal cancers, they help contextualize the potential relevance of dietary patterns during survivorship.

#### Post-treatment diet quality

Following cancer treatment, a substantial proportion of survivors exhibit suboptimal dietary patterns, frequently characterized by reduced diet quality and a meaningful intake of UPFs. Although direct evidence linking post-treatment UPF consumption to cancer recurrence remains limited, an expanding body of literature suggests that dietary patterns in survivorship may be associated with long-term outcomes, including symptom burden, metabolic health, quality of life, and competing mortality.

Several studies consistently show that diet quality tends to deteriorate after cancer treatment, with a shift toward more processed and energy-dense foods. In colorectal cancer (CRC) patients with newly formed colostomies, a prospective study demonstrated a significant decline in diet quality over a 6-month period, with increased intake of energy, lipids, and sodium, alongside reduced consumption of protein, fiber, and essential micronutrients such as vitamins B1 and C. Notably, intake of minimally processed foods and fruits decreased, while UPF consumption increased, resulting in a worsening of overall healthy eating index scores (19). Similar trends have been observed in broader survivorship populations. Adult cancer survivors frequently report difficulties in maintaining a healthy diet and body weight, alongside a need for more structured nutritional guidance (20).

In 396 stage I–III CRC survivors followed 6 weeks−24 months post-treatment, higher post-treatment UPF intake (and sugar-sweetened drinks, higher energy density) was longitudinally associated with worse health-related quality of life, more fatigue, and more chemotherapy-induced peripheral neuropathy symptoms (21). These findings do not establish an effect on recurrence or survival but suggest that diet quality may be relevant to recovery, symptom persistence, and functional outcomes after treatment.

In broader cancer-survivor populations, indirect evidence also supports a possible association between UPF exposure and adverse long-term outcomes. In 799 Italian adults with prior cancer, each 5% higher UPF proportion in the diet was linked to 14% higher premature mortality, independent of overall diet quality. Notably, systemic inflammation (measured by C-reactive protein) and autonomic dysfunction (resting heart rate) accounted for approximately half of this association, suggesting that UPF-related inflammatory pathways may contribute to both cancer progression and mortality risk (22). In Lebanese cancer survivors in remission, UPFs represented a relatively low proportion of food weight but contributed substantially to total energy intake (17.5%) and were associated with higher sugar, fat, and saturated fat intake and lower vitamin C adequacy (23). These patterns may contribute to metabolic and inflammatory conditions that are relevant to long-term survivorship, although direct evidence linking them to recurrence or secondary malignancies remains limited.

#### Western dietary patterns

Western dietary patterns were classified as indirect evidence, as they substantially overlap with UPF consumption while representing a broader dietary construct rather than a direct measure of food processing. These patterns typically include high intake of processed meats, refined grains, sweets, caloric beverages, convenience foods, sauces, saturated fats, and energy-dense products, with low intake of whole grains, fiber-rich foods, fruits, and vegetables. This nutritional profile may promote obesity, insulin resistance, chronic systemic inflammation, and gut microbiota alterations, all of which are biologically relevant to cancer development and survivorship.

Epidemiological data strongly support the association between Western dietary patterns and adverse CRC outcomes. In the EPIC-Spain cohort, adherence to a Western diet was associated with increased CRC risk, particularly within the first 10 years of follow-up (HR 1.17, 95% CI 0.99–1.37), with stronger effects observed in females and in rectal cancers (24). In contrast, higher adherence to a Mediterranean dietary pattern was associated with reduced colorectal cancer risk, particularly for distal colon cancer (HR 0.84, 95% CI 0.73–0.98) (24). The stronger associations observed for distal and rectal tumors suggest a role of local luminal exposure to diet-derived carcinogens, which may also influence tumor aggressiveness and recurrence risk through sustained epithelial damage and inflammation. These findings support the broader concept that dietary pattern quality may influence colorectal carcinogenesis, although they do not directly address post-diagnostic outcomes.

Evidence from large prospective cohorts suggests that the relationship between dietary patterns and colorectal cancer may differ according to sex. In the EPIC-Spain cohort, adherence to a Western dietary pattern was more strongly associated with increased colorectal cancer risk among women, particularly for rectal cancer and during the first decade of follow-up (24). In contrast, several analyses from the broader EPIC cohort reported stronger associations between pro-inflammatory dietary patterns (dietary profiles with higher inflammatory potential, typically characterized by higher intake of red and processed meats, refined grains, added sugars, and lower intake of fruits, vegetables, whole grains, and fiber) and colorectal cancer risk in men, whereas associations were less consistent in women (25, 26). These differences may reflect the influence of sex hormones, inflammatory responses, and baseline lifestyle characteristics, with estrogens potentially exerting a protective effect that modifies diet–cancer associations in women (26). These differences may reflect hormonal, inflammatory, behavioral, and lifestyle-related factors. Importantly, no major sex-specific differences were reported in the only available study evaluating post-diagnostic UPF intake and colorectal cancer survivorship (1). Overall, sex- and age-specific analyses remain inconsistently reported across studies, limiting the assessment of potential effect modification and highlighting an important area for future research.

Although Western dietary patterns and UPF exposure overlap, they should not be considered interchangeable. Western dietary patterns describe an overall dietary profile, whereas the NOVA classification categorizes foods according to the nature, extent, and purpose of industrial processing. Therefore, Western diet studies were interpreted as contextual evidence supporting biological plausibility rather than as direct evidence of post-diagnostic UPF effects.

#### Lifestyle intervention

Lifestyle intervention studies were included as indirect evidence when they addressed dietary quality, UPF reduction, or broader behavioral modification in survivorship or related populations. Reducing UPF consumption represents a major public health challenge because these products are highly palatable, aggressively marketed, inexpensive, and linked to hedonic and addictive-like eating behaviors (27, 28). Psychological factors such as stress, anxiety, depression, and food addiction traits further contribute to sustained UPF consumption and may reduce adherence to dietary interventions, particularly among vulnerable populations such as cancer survivors (27).

Current evidence suggests that standard nutritional counseling alone often produces limited and short-lived effects on UPF reduction (29, 30). In contrast, more intensive educational and behavioral approaches—including structured meal planning, group-based interventions, and practical strategies to identify and replace UPFs—appear more promising and may substantially reduce UPF intake in motivated individuals (31). However, the long-term sustainability of these interventions remains uncertain, as benefits frequently decline without continuous support and favorable environmental conditions (30, 31).

Beyond individual counseling, broader institutional and policy-level strategies are increasingly being explored. These include taxation of sugary beverages and snacks, front-of-package warning labels, restrictions on advertising to children, and school-based nutritional policies (32, 33). Although many of these measures show potential benefit, implementation remains inconsistent and insufficiently supported at a population level. Most current policies focus primarily on consumer awareness rather than structural changes targeting food marketing, affordability, and accessibility of healthier alternatives.

Lifestyle modification also extends beyond dietary habits. Physical activity is an important component of cancer survivorship, with beneficial effects on treatment-related symptoms, physical function, metabolic health, and long-term outcomes. The CHALLENGE trial demonstrated that a structured exercise program after adjuvant chemotherapy significantly improved disease-free survival in colon cancer survivors, reinforcing the concept that modifiable lifestyle factors may influence prognosis after treatment (34). While this evidence does not directly address UPF exposure, it supports the broader relevance of lifestyle medicine in survivorship care.

Direct and indirect survivorship studies are summarized in Table 1.

**Table 1:** Direct and indirect survivorship studies evaluating post-diagnostic diet, UPF exposure, or dietary quality in cancer survivors.

Level of evidence	Study	Cancer type/Population	Study design	Sample size	Timing	Exposure assessed	Outcomes	Key findings	Main limitations
Direct post-diagnostic GI cancer evidence	Hang et al. (1)	Stage I–III colorectal cancer survivors	Prospective cohort	2,498	Post-diagnosis (≥6 months after diagnosis)	UPF intake	Overall mortality, CRC-specific mortality, cardiovascular mortality	Higher UPF intake associated with increased cardiovascular mortality; no significant association with overall or CRC-specific mortality; some UPF subgroups associated with worse CRC-specific mortality	Observational design; self-reported dietary assessment
Indirect GI survivorship evidence	Kenkhuis et al. (21)	Stage I–III colorectal cancer survivors	Longitudinal cohort	396	6 weeks−24 months post-treatment	UPF intake, sugar-sweetened beverages, energy density	Quality of life, fatigue, chemotherapy-induced peripheral neuropathy	Higher UPF intake associated with worse HRQoL, greater fatigue, and neuropathy symptoms	No survival endpoints; symptom-focused outcomes
Indirect post-treatment dietary-quality evidence	Duarte et al. (19)	Colorectal cancer patients with colostomy	Prospective study	60	Post-surgery follow-up	Diet quality and UPF consumption	Nutritional quality, dietary pattern changes	Increased UPF intake and worsening dietary quality over time after surgery	Small sample size; short follow-up; no oncologic endpoints; single-center design
Indirect mixed cancer-survivor evidence	Bonaccio et al. (22)	Adult cancer survivors	Prospective cohort	799	Post-diagnosis	Dietary proportion of UPFs	Premature mortality	Each 5% increase in UPF intake associated with 14% higher premature mortality	observational design; possible residual confounding; limited site-specific outcome data.
Indirect mixed cancer-survivor evidence	Helou et al. (23)	Cancer survivors in remission	Cross-sectional study	198	Survivorship phase	UPF intake and nutrient profile	Nutritional adequacy	Higher UPF intake associated with increased sugar/fat intake and lower vitamin C adequacy	Cross-sectional design; no survival or recurrence outcomes; limited ability to infer temporality
Indirect survivorship nutritional-behavior evidence	O'Callaghan et al. (20)	Adult cancer survivors	Cross-sectional survey	170	Survivorship phase	Dietary quality and nutritional behaviors	Nutritional needs and lifestyle patterns	Survivors reported suboptimal diet quality and need for nutritional support	Cross-sectional survey; self-reported behaviors; no survival or recurrence endpoints.
Interventional feasibility evidence	HEAL ABC Trial – Sremanakova et al. (18)	Bowel cancer survivors	Feasibility randomized trial	72	Post-treatment	Healthy eating and lifestyle intervention	Feasibility, lifestyle modification	Lifestyle interventions feasible in CRC survivorship care	Feasibility trial; small sample size; not powered for recurrence or survival endpoints
Contextual dietary-intervention evidence	Jameson et al. (36)	Metastatic pancreatic cancer	Randomized phase II trial	162	During treatment	Ketogenic dietary intervention	Treatment response, survival	Demonstrates increasing interest in dietary modulation during systemic therapy	dietary intervention not UPF-specific; metastatic setting; ketogenic intervention; limited relevance to post-diagnostic UPF exposure

CRC, colorectal cancer; GI, gastrointestinal; HR, hazard ratio; CI, confidence interval; HRQoL, health-related quality of life; CVD, cardiovascular disease; UPF, ultra-processed food; QoL, quality of life.

### Contextual epidemiological evidence by cancer site

Because post-diagnostic evidence was unavailable for most gastrointestinal cancer sites, incidence-based epidemiological studies were summarized as contextual evidence. These studies do not establish prognostic effects after diagnosis, but they help identify cancer sites in which UPF exposure or related dietary patterns may be biologically and clinically relevant.

#### Gastric cancer

No post-diagnostic studies evaluating UPF consumption and clinical outcomes in gastric cancer were identified. However, epidemiological evidence suggests a potential association between UPF exposure and gastric carcinogenesis. A recent meta-analysis of prospective cohorts reports that higher UPF intake was associated with an increased risk of non-cardia gastric cancer, supporting a potential role of processed dietary exposures in upper gastrointestinal malignancies (19). Similarly, a multicenter Spanish case–control study reported a positive association between UPF consumption and stomach cancer risk (17).

Several mechanisms may explain these observations. UPFs frequently contain salt, nitrites, nitrates, advanced glycation end-products, and compounds generated during industrial processing that may contribute to gastric mucosal injury, oxidative stress, and chronic inflammation. Moreover, diets characterized by high UPF consumption are typically low in fiber, fruits, and vegetables, potentially reducing exposure to protective micronutrients and bioactive compounds (13, 20).

Given the established importance of nutritional status in patients with gastric cancer, particularly in relation to treatment tolerance, sarcopenia, and postoperative outcomes, understanding the impact of post-diagnostic UPF exposure represents an important area for future research. However, direct evidence linking UPF consumption after diagnosis with recurrence or survival remains unavailable.

#### Pancreatic cancer

No studies specifically evaluating post-diagnostic UPF consumption and survival outcomes in pancreatic cancer were identified. Nevertheless, several epidemiological investigations support a potential role of dietary exposures in pancreatic carcinogenesis. In the PLCO Cancer Screening Trial, participants with the highest UPF consumption had a 49% higher risk of pancreatic cancer compared with those in the lowest category (HR 1.49, 95% CI 1.07–2.07), with evidence of a dose–response relationship (21). Similar findings were reported in a subsequent systematic review and meta-analysis, although the pancreatic cancer estimate was largely driven by this single prospective cohort (9).

Additional evidence supports the broader relevance of diet in pancreatic cancer. A recent meta-analysis of 19 prospective cohorts reported that the highest intake of total red meat was associated with a 12% increased risk of pancreatic cancer, while diets rich in fruits, vegetables, nuts, and whole grains appeared protective (22). The authors estimated that a substantial proportion of pancreatic cancers may be attributable to unfavorable dietary exposures and highlighted several biologically plausible mechanisms, including chronic inflammation, oxidative stress, insulin resistance, activation of the IGF-1 pathway, and exposure to carcinogenic compounds generated during food processing and high-temperature cooking (22).

However, evidence remains inconsistent. A multicenter Spanish case–control study did not observe a significant association between total UPF intake and pancreatic cancer, despite reporting positive associations for oesophageal and gastric cancers (17). Likewise, a recent meta-analysis of prospective cohorts found significant associations between UPF consumption and colorectal and non-cardia gastric cancers, but not pancreatic cancer (19). Overall, current evidence suggests that dietary exposures may contribute to pancreatic cancer development, but direct data evaluating whether post-diagnostic UPF consumption influences treatment outcomes, recurrence, or survival are currently lacking.

Existing dietary intervention studies in pancreatic cancer generally assess broader nutritional or metabolic strategies rather than UPF exposure specifically. For example, recent intervention studies evaluating ketogenic or metabolic-targeted dietary approaches during pancreatic cancer treatment reflect growing interest in dietary modulation as a supportive component of therapy, but they cannot be interpreted as direct evidence on UPF exposure (23).

#### Esophageal cancer

No studies evaluating post-diagnostic UPF consumption and oncologic outcomes in oesophageal cancer were identified. Nevertheless, indirect epidemiological evidence suggests that UPF intake may contribute to oesophageal carcinogenesis. In the multicenter Spanish case–control study, higher UPF consumption was associated with increased risk of esophageal cancer, supporting previous observations linking Western dietary patterns with upper gastrointestinal malignancies (17).

Potential mechanisms include chronic exposure to processing-derived compounds, nitrosamines, oxidative stress, and low dietary fiber intake, which may promote chronic mucosal inflammation and carcinogenesis. In addition, obesity and gastroesophageal reflux disease, both strongly associated with Westernized dietary patterns and UPF consumption, are recognized risk factors for esophageal adenocarcinoma (3, 20).

Although nutritional impairment and weight loss are highly prevalent in patients with esophageal cancer and significantly affect treatment outcomes, no studies have specifically evaluated whether post-diagnostic UPF exposure influences recurrence, treatment response, or survival. Further prospective research is warranted.

#### Liver cancer

No direct studies evaluating post-diagnostic UPF consumption in hepatocellular carcinoma, cholangiocarcinoma, or other biliary tract cancers were identified. Evidence regarding cancer incidence is also more limited than for colorectal or gastric cancer. However, several biological pathways associated with UPF consumption, including obesity, insulin resistance, metabolic syndrome, metabolic dysfunction-associated steatotic liver disease, and chronic systemic inflammation, are recognized drivers of hepatocarcinogenesis (20).

Emerging epidemiological studies have linked higher UPF consumption to increased risk of obesity-related and liver-related outcomes, suggesting that hepatobiliary malignancies may be indirectly influenced through metabolic dysfunction and liver disease progression. Given the growing global burden of metabolic dysfunction-associated steatotic liver disease and its role as a precursor of hepatocellular carcinoma, dietary quality may become increasingly relevant in hepatobiliary cancer prevention and survivorship (24, 25). However, evidence on post-diagnostic UPF exposure and outcomes in hepatobiliary cancers remains absent.

### Mechanistic and translational evidence

**Figure 2:**
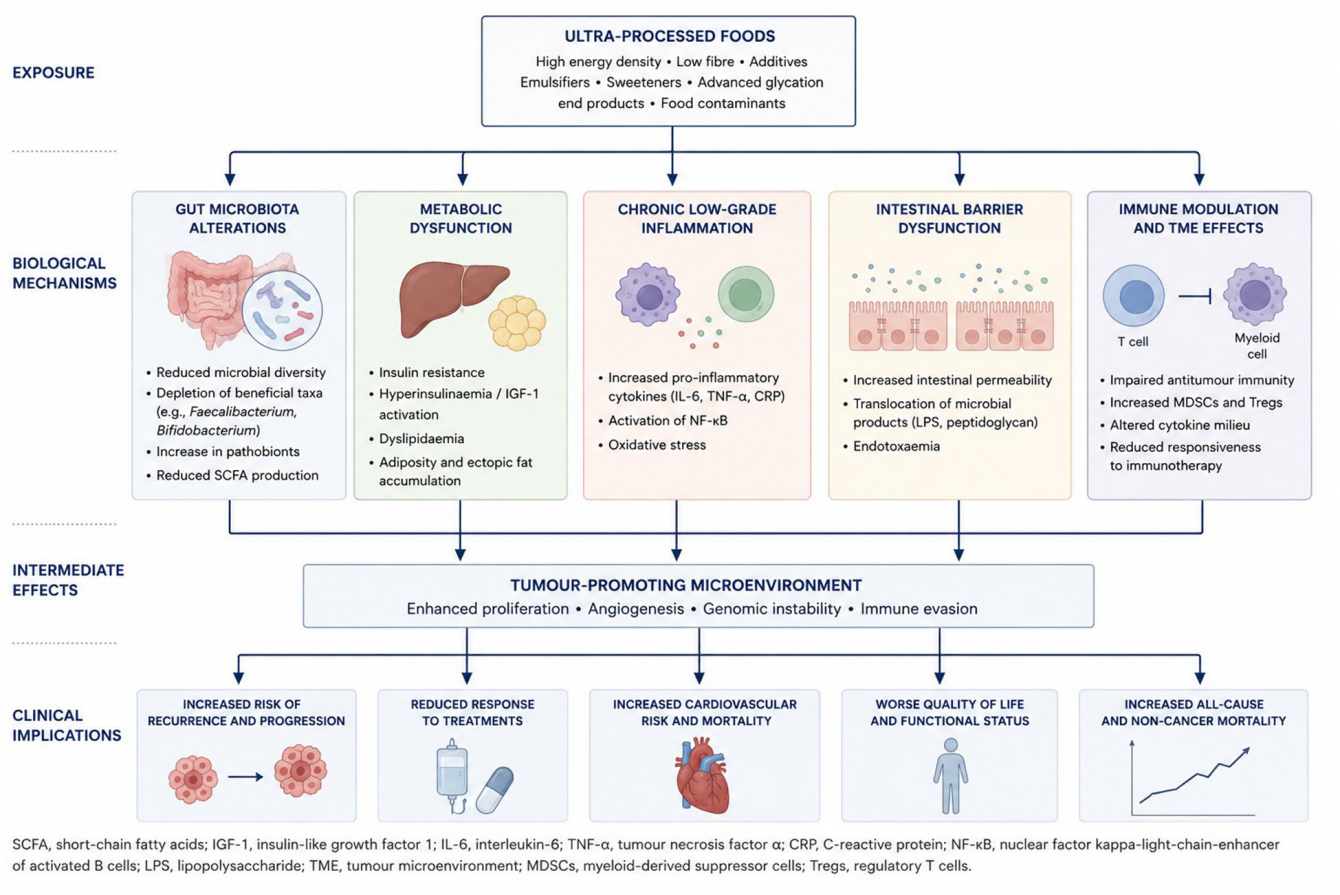
Proposed biological mechanisms linking ultra-processed food exposure with gastrointestinal cancer progression and survivorship outcomes. Ultra-processed foods may contribute to metabolic dysfunction, chronic inflammation, gut microbiota alterations, and exposure to additives or processing-related compounds. These pathways provide biological plausibility for potential effects on gastrointestinal cancer survivorship, although direct post-diagnostic clinical evidence remains limited. Current evidence in colorectal cancer survivors suggests that competing risks, including cardiovascular mortality, may be particularly relevant. This figure was generated using the OpenAI image generation tool / DALL·E-based image generation tool and the input prompt can be found in Supplementary File 1.

Mechanistic and translational evidence was considered separately from clinical outcome studies. These data were used to explore biological plausibility rather than to infer causality or clinical effectiveness. The main pathways potentially linking UPF exposure to gastrointestinal cancer development and survivorship include metabolic dysregulation, chronic inflammation, microbiota disruption, and exposure to additives or processing-related compounds. The proposed biological mechanisms linking UPF exposure with gastrointestinal cancer progression and survivorship outcomes are summarized in Figure 2.

Ultra-processed foods may influence clinical outcomes in gastrointestinal cancer survivors through inflammatory, metabolic, microbiota-mediated, and additive-related pathways. Current evidence suggests a stronger impact on cardiovascular mortality than on cancer-specific outcomes, highlighting the role of competing risks.

#### Metabolic dysregulation

UPFs are typically energy-dense and nutritionally poor, promoting weight gain, obesity, and metabolic syndrome (11). Obesity is a well-established risk factor for multiple cancers and may account for a substantial proportion of diet-related cancer burden (10). Mechanistically, excess adiposity leads to chronic hyperinsulinemia and activation of insulin and pro-growth pathways (AKT/mTOR), which stimulate cellular proliferation and inhibit apoptosis (10). Additionally, altered adipokine secretion, sex hormones, fatty acid metabolism, extracellular matrix remodeling, and immune dysregulation create a pro-tumorigenic systemic environment that supports tumor initiation and progression (12).

#### Chronic inflammation and oxidative stress

UPFs promote low-grade chronic inflammation, with raised pro-inflammatory cytokines (e.g., IL-6, TNF-α) and oxidative stress, influencing every step of carcinogenesis from initiation to metastasis, contributing to DNA damage, genomic instability, and tumor-promoting microenvironments (12). *In vitro* and experimental models show UPF components (emulsifiers, processed food digests) damaging epithelial tight junctions and enhancing epithelial–immune inflammatory crosstalk (11, 13). Persistent inflammation may also impair immune surveillance, favoring tumor initiation and progression. However, these mechanistic data should be interpreted as supportive rather than direct clinical evidence.

#### Gut microbiome alterations

UPF consumption has been associated with alterations in gut microbiota composition and function, including reduced microbial diversity and enrichment of pro-inflammatory bacterial taxa (12). Diet-induced dysbiosis may impair intestinal barrier integrity, promote systemic inflammation, and increase exposure to microbiota-derived metabolites involved in gastrointestinal carcinogenesis. Western dietary patterns and high UPF intake are also associated with depletion of short-chain fatty acid (SCFA)-producing bacteria and reduced production of metabolites such as butyrate, which contribute to epithelial integrity and immune homeostasis.

These alterations may influence not only carcinogenesis but also therapeutic response, particularly in the context of immunotherapy (15, 16, 35). Emerging evidence suggests that microbiome composition may influence responsiveness to immune checkpoint inhibitors and other systemic therapies (15, 16). In parallel, antibiotic-induced disruption of gut microbial communities has been linked to reduced efficacy of immune checkpoint inhibitors and poorer oncologic outcomes, further supporting the relevance of host–microbiome interactions in cancer therapy (35).

Although direct evidence linking UPF-driven dysbiosis to clinical outcomes in gastrointestinal cancer survivors remains limited, current translational data support a biologically plausible role of the gut microbiota as a mediator between dietary exposure, systemic inflammation, and therapeutic response.

#### Food additives and processing-related compounds

UPFs are a major source of non-nutritional exposures that may contribute directly to carcinogenesis. Industrial processing generates neoformed contaminants such as acrylamide, heterocyclic amines, and advanced glycation end-products, many of which have recognized carcinogenic properties (12). In addition, food additives (e.g., emulsifiers, sweeteners, colorants) and packaging-derived chemicals (e.g., bisphenols) may exert endocrine-disrupting and genotoxic effects (17). In fact, nitrites, emulsifiers, artificial sweeteners, acrylamide, bisphenols, and phthalates promote oxidative stress, microbiota disruption, inflammatory signaling, endocrine dysregulation, and DNA damage. These exposures may induce epigenetic alterations and disrupt hormonal and metabolic signaling pathways, further promoting tumorigenesis (11). These mechanisms support biological plausibility but remain insufficient to infer direct prognostic effects after cancer diagnosis.

Contextual epidemiological and mechanistic evidence is summarized in Table 2.

**Table 2:** Contextual epidemiological and mechanistic evidence supporting biological plausibility of UPF-related gastrointestinal cancer outcomes.

Evidence type	Study	Cancer type/outcome	Main findings	Relevance to this review	Interpretation/major limitations
Incidence-based cohort study	Wang et al. (2)	Colorectal cancer incidence	Higher UPF intake associated with increased CRC risk	Strong epidemiological rationale for biological plausibility	Contextual evidence only; no post-diagnostic exposure assessment; cannot inform recurrence, treatment response, or survivorship outcomes.
Incidence-based cohort study	Fiolet et al. (5)	Overall cancer incidence	Increased cancer risk associated with UPF intake	Supports carcinogenic potential of UPFs	General population cohort; incidence rather than survivorship outcomes; not specific to gastrointestinal cancers.
Systematic review and meta-analysis	Meine et al. (14)	Gastrointestinal cancer risk	Meta-analysis confirmed increased GI cancer risk with higher UPF intake	Reinforces consistency of epidemiological signal	Based mainly on observational incidence studies; heterogeneity across cohorts, dietary assessment methods, and UPF definitions; no direct post-diagnostic outcomes.
Systematic review and meta-analysis	Shu et al. (3)	Colorectal cancer risk	Positive association between UPF intake and CRC risk	Supports role of UPF exposure to colorectal carcinogenesis.	Incidence-focused evidence; variability in UPF classification and dietary assessment; cannot be extrapolated directly to prognosis after diagnosis.
Incidence-based cohort study	Al Nahas et al. (8)	Colorectal cancer risk	Higher degree of food processing associated with increased CRC risk	Suggests processing level itself may influence carcinogenesis	Association study focused on incidence; limited relevance to survivorship; no assessment of post-diagnostic UPF exposure.
Competing-risk observational cohort	Campanella et al. (7)	GI cancer mortality	Higher UPF intake associated with increased GI cancer mortality	Suggests a possible association between UPF exposure and gastrointestinal cancer mortality at the population level.	Contextual evidence; limited post-diagnostic assessment; geographic and population-specific cohort; residual confounding cannot be excluded.
Mixed cancer-survivor cohort	Zhao et al. (6)	Mortality among cancer survivors	Higher UPF intake associated with increased all-cause and cancer mortality	Supports relevance of post-diagnostic diet in survivorship	Indirect evidence; mixed cancer-survivor population; not specific to gastrointestinal malignancies; observational design; possible lifestyle confounding.
Prospective cohort study	Kliemann et al. (37)	Multi-site cancer incidence	Replacing UPFs with minimally processed foods was associated with lower cancer risk across several sites.	Supports the potential relevance of food processing level beyond nutrient composition alone.	Incidence-based evidence; substitution models are observational; no post-diagnostic assessment or survivorship endpoints.
Dietary-pattern cohort study	Castelló et al. (24)	Colorectal cancer risk	Western dietary pattern was associated with higher colorectal cancer risk, whereas Mediterranean dietary pattern was associated with lower risk.	Provides indirect evidence linking broader dietary patterns overlapping with UPF exposure to colorectal carcinogenesis.	Western diet is not equivalent to NOVA-defined UPF exposure; incidence-focused; indirect relevance to post-diagnostic survivorship outcomes.
Mechanistic/translational evidence	Kliemann et al. (11); Babalola et al. (12)	Biological mechanisms	UPFs may promote metabolic dysfunction, inflammation, oxidative stress, microbiota alterations, and exposure to additives or processing-related compounds.	Provides biological plausibility for potential effects of UPF exposure on gastrointestinal cancer development and survivorship.	Mechanistic evidence only; does not establish clinical prognostic impact after cancer diagnosis.

CRC, colorectal cancer; GI, gastrointestinal; HRQoL, health-related quality of life; HR, hazard ratio; UPF, ultra-processed food.

This table summarizes contextual epidemiological and mechanistic evidence. These studies were not treated as direct post-diagnostic prognostic evidence. Limitations reflect the relevance of each source to the review question and should not be interpreted as a formal risk-of-bias assessment.

### Clinical implications

The clinical implications of the available evidence should be interpreted cautiously. Direct evidence linking post-diagnostic UPF consumption to gastrointestinal cancer-specific outcomes remains scarce, and no randomized trial has yet demonstrated that reducing UPF intake improves recurrence, survival, or treatment response. Nevertheless, the available data suggest that UPF exposure may be relevant to survivorship through its association with diet quality, symptom burden, metabolic health, cardiovascular risk, and overall nutritional adequacy.

From a clinical perspective, these findings support the inclusion of dietary quality and food-processing level as exploratory dimensions in nutritional assessment and survivorship research. In colorectal cancer survivors, post-treatment dietary deterioration and higher UPF-related dietary exposures have been associated with poorer quality of life, fatigue, neuropathy symptoms, and cardiovascular mortality. These findings do not establish causality, but they indicate that dietary patterns after treatment may contribute to broader survivorship outcomes beyond cancer recurrence alone.

At present, UPF reduction should be considered a plausible and testable survivorship strategy rather than an evidence-based intervention with proven oncologic benefit. Future clinical programs should evaluate whether structured nutritional counseling, behavioral support, and broader lifestyle interventions can sustainably reduce UPF intake and whether such changes translate into improvements in metabolic, cardiovascular, quality-of-life, and oncologic outcomes.

## Discussion

This scoping review mapped the available evidence on post-diagnostic ultra-processed food (UPF) exposure in gastrointestinal cancer populations. The main finding is that the evidence base remains limited, heterogeneous, and unevenly distributed across cancer sites, with direct post-diagnostic evidence largely restricted to colorectal cancer survivors. For gastric, pancreatic, oesophageal, and hepatobiliary cancers, available evidence was predominantly indirect, derived from incidence-based epidemiology, broader dietary-pattern research, mixed cancer-survivor cohorts, or mechanistic and translational studies. These sources support biological plausibility and help identify research gaps but should not be interpreted as direct evidence of prognostic impact after diagnosis.

The only directly relevant prospective study identified in colorectal cancer survivors reported that higher post-diagnostic UPF intake was associated with increased cardiovascular mortality, but not with overall or colorectal cancer-specific mortality. This finding highlights the importance of competing risks in survivorship and suggests that the potential clinical relevance of UPF exposure may extend beyond cancer-specific endpoints. However, the observational design, reliance on self-reported dietary assessment, and potential residual confounding preclude causal inference.

A second key finding is the heterogeneity of exposure definitions. Only a limited number of studies assessed UPF intake using NOVA-based or NOVA-compatible definitions. Several relevant studies evaluated broader dietary constructs, including Western dietary patterns, post-treatment diet quality, fast food intake, sugar-sweetened beverages, or lifestyle interventions. Although these exposures overlap with UPF consumption, they are not interchangeable. This heterogeneity limits comparability across studies and reinforces the need for standardized dietary assessment in future survivorship research.

The distribution of outcomes was also uneven. Mortality, cardiovascular mortality, quality of life, fatigue, and nutritional adequacy were the most frequently represented outcomes, whereas recurrence, progression, treatment response, treatment tolerance, and cancer-specific survival remain poorly studied. In particular, no prospective post-diagnostic cohorts were identified for most gastrointestinal cancer sites, and no randomized trials specifically testing UPF reduction on oncologic outcomes were available. Therefore, current evidence is insufficient to determine whether UPF exposure influences cancer progression, recurrence, or response to systemic therapy.

Mechanistic and translational studies provide plausible pathways linking UPF exposure with metabolic dysfunction, chronic inflammation, oxidative stress, gut microbiota alterations, and exposure to additives or processing-related compounds (10–14). These pathways are biologically relevant to gastrointestinal cancer biology and survivorship, particularly in relation to metabolic health and cardiovascular comorbidity. However, mechanistic plausibility should not be interpreted as proof of clinical effectiveness of UPF reduction after cancer diagnosis.

The available evidence has cautious clinical implications. Nutritional assessment is already relevant in gastrointestinal oncology because of its relationship with treatment tolerance, body composition, symptom burden, cardiovascular risk, and quality of life. The findings of this review suggest that UPF exposure may be a useful dimension to capture in future dietary assessment and survivorship research. However, evidence is not yet sufficient to recommend UPF reduction as a strategy specifically proven to improve recurrence-free survival, cancer-specific survival, or treatment response in gastrointestinal cancer patients.

This review has several limitations. First, the structured database search was limited to PubMed/MEDLINE and supplemented by manual reference screening; therefore, relevant studies indexed exclusively in other bibliographic databases may have been missed. Second, substantial heterogeneity existed across studies regarding dietary assessment methods, UPF definitions, timing of exposure evaluation, and outcomes assessed, limiting direct comparability. Third, much of the available evidence was indirect or contextual, and only a small number of studies directly addressed post-diagnostic UPF exposure in gastrointestinal cancer populations. Fourth, most clinical evidence was observational and relied on self-reported dietary intake, which is subject to measurement error and residual confounding. Finally, because of the limited number and heterogeneity of eligible studies, quantitative synthesis was not appropriate.

Overall, this scoping review identifies post-diagnostic UPF exposure as an underexplored area in gastrointestinal cancer survivorship. The current evidence does not establish causality or clinical effectiveness of UPF reduction, but it supports the need for prospective cohorts and intervention studies using standardized UPF assessment, repeated post-diagnostic dietary measurements, clinically relevant oncologic and survivorship endpoints, and integration of metabolic, inflammatory, and microbiome biomarkers.

Beyond individual behavior, the pervasive availability and marketing of UPFs represent major barriers to sustained dietary modification. Although policy-level interventions such as taxation, warning labels, and restrictions on advertising have shown potential benefit, implementation remains limited and heterogeneous across healthcare system (32, 33). Similarly, educational and behavioral interventions often lose effectiveness over time in the absence of continuous support and environmental change.

Beyond the specific question of ultra-processed food consumption, this review highlights a broader issue: lifestyle factors remain substantially underutilized in contemporary oncology despite their recognized role in chronic disease prevention and survivorship. Diet quality, physical activity, body composition, and other behavioral factors are increasingly acknowledged as modifiable determinants of both cancer incidence and long-term outcomes after diagnosis. The recent CHALLENGE trial demonstrated that a structured exercise intervention can improve disease-free survival in patients treated for colon cancer, providing direct evidence that lifestyle modification may influence prognosis even after completion of standard oncologic therapies. From a public health perspective, promoting healthier dietary patterns and physical activity has the potential not only to reduce the burden of cancer and other chronic diseases, but also to improve quality of life, decrease treatment-related morbidity, and potentially reduce healthcare costs associated with long-term survivorship. Nevertheless, nutritional assessment and lifestyle counseling remain inconsistently integrated into routine oncology practice, highlighting an important gap between emerging evidence and clinical implementation.

### Future directions

Despite increasing epidemiological and mechanistic evidence linking UPFs to gastrointestinal carcinogenesis, major gaps remain regarding the impact of post-diagnostic UPF exposure on long-term outcomes after cancer diagnosis. Future research should move beyond incidence-based analyses and prioritize prospective survivorship cohorts with repeated dietary assessments after diagnosis, during treatment, and throughout follow-up.

First, standardized assessment of UPF exposure is needed. Future studies should apply NOVA-based or clearly NOVA-compatible definitions, report UPF intake as both absolute intake and proportion of total energy intake, and distinguish total UPF exposure from specific UPF subgroups. This is particularly important because available evidence suggests that different UPF categories may have different associations with cardiovascular and cancer-specific outcomes.

Second, future cohorts should include clinically relevant endpoints beyond all-cause mortality. These should include recurrence, progression, cancer-specific survival, cardiovascular mortality, treatment tolerance, symptom burden, body composition, quality of life, and competing risks. Such endpoints are especially important in gastrointestinal cancer survivorship, where malnutrition, sarcopenia, metabolic dysfunction, and cardiovascular comorbidity may interact with oncologic outcomes.

Third, translational studies should integrate dietary assessment with microbiome profiling, inflammatory biomarkers, metabolomics, insulin/IGF-1 signaling, immune signatures, and treatment-response data. This approach may help clarify whether UPF exposure influences survivorship primarily through metabolic and cardiovascular pathways, tumor-related mechanisms, microbiota-mediated effects, or a combination of these processes.

Fourth, randomized lifestyle intervention trials are needed to determine whether reducing UPF intake and promoting minimally processed dietary patterns can be achieved and sustained in gastrointestinal cancer survivors. These trials should evaluate not only dietary adherence and metabolic outcomes, but also quality of life, treatment-related symptoms, cardiovascular risk, recurrence, and survival. Until such data are available, UPF reduction should be considered a plausible and testable survivorship strategy rather than a proven oncologic intervention.

Clarifying the role of post-diagnostic UPF exposure may ultimately support the integration of standardized nutritional assessment and evidence-based dietary strategies into gastrointestinal cancer survivorship care.

## Conclusions

Evidence regarding post-diagnostic ultra-processed food exposure in gastrointestinal cancers remains limited, heterogeneous, and predominantly observational. Direct evidence is currently largely restricted to colorectal cancer survivors and suggests a possible association between higher UPF intake and cardiovascular mortality, while evidence for cancer-specific outcomes remains inconclusive.

For other gastrointestinal cancer sites, available data are mainly indirect or contextual and derive from incidence-based epidemiology, broader dietary-pattern research, and mechanistic studies. These findings support biological plausibility but do not establish a prognostic effect of UPF exposure after diagnosis.

Future prospective cohorts and intervention trials are needed to determine whether reducing UPF intake after diagnosis can improve metabolic health, treatment tolerance, quality of life, competing mortality, recurrence, or survival in gastrointestinal cancer patients. Until such evidence is available, UPF exposure should be considered an important research priority and a potential component of comprehensive survivorship assessment, rather than a proven therapeutic target.
