# Supplementary material for: Post-diagnostic ultra-processed food exposure in gastrointestinal cancers: scoping review with narrative synthesis and clinical implications
Source: Front Nutr. 2026 Jul 14;13:1884359. doi: 10.3389/fnut.2026.1884359 (PMC13410676; doi:10.3389/fnut.2026.1884359)
Supplement: Supplementary file 1 [file Table_1.docx]

**Supplementary Table S1. PRISMA-ScR checklist**

| Item | PRISMA-ScR checklist item | Relevant manuscript section |
| --- | --- | --- |
| Title | Identify the report as a scoping review. | Title page |
| Abstract | Provide a structured summary including background, objectives, eligibility criteria, sources of evidence, charting methods, results, and conclusions that relate to the review questions and objectives. | Abstract |
| Rationale | Describe the rationale for the review in the context of what is already known, and explain why a scoping review approach was appropriate. | Introduction; Methods, Study design and review framework |
| Objectives | Provide an explicit statement of the questions and objectives being addressed with reference to key elements such as population, concept, and context. | Introduction; Methods, Study design and review framework |
| Eligibility criteria | Specify characteristics of sources of evidence used as eligibility criteria and provide a rationale. | Methods, Eligibility criteria |
| Information sources | Describe all information sources and the date of the most recent search. | Methods, Search strategy and information sources; Supplementary Table S2 |
| Search | Present the full electronic search strategy for at least one database, including limits used, so that it can be repeated. | Supplementary Table S2 |
| Selection of sources of evidence | State the process for selecting sources of evidence included in the scoping review. | Methods, Study selection process; Figure 1 |
| Data charting process | Describe the methods of charting data from included sources and any processes for obtaining and confirming data. | Methods, Data extraction |
| Synthesis of results | Describe the methods used for handling and summarizing the charted data. | Methods, Data extraction; Methods, Evidence hierarchy |
| Synthesis of results | Describe methods of handling and summarizing data | Methods – Data extraction; Results |
| Results of individual sources | Present relevant data from included sources | Results; Table 1; Table 2 |
| Summary of evidence | Summarize the main results, including how they relate to the review questions and objectives. | Discussion |
| Limitations | Discuss limitations of the scoping review process. | Discussion |
| Conclusions | Provide a general interpretation of the results with respect to the review questions and objectives, as well as potential implications and next steps. | Clinical implications; Future directions; Conclusions |
| Funding | Describe sources of funding for the included sources of evidence and for the scoping review, and the role of funders. | Funding; Conflict of Interest |
