# Supplementary material for: Post-diagnostic ultra-processed food exposure in gastrointestinal cancers: scoping review with narrative synthesis and clinical implications
Source: Front Nutr. 2026 Jul 14;13:1884359. doi: 10.3389/fnut.2026.1884359 (PMC13410676; doi:10.3389/fnut.2026.1884359)
Supplement: Supplementary file 2 [file Table_2.docx]

**Supplementary Table S2. PubMed/MEDLINE search strategy**

| Component | Search terms |
| --- | --- |
| Database searched | PubMed/MEDLINE |
| Search period | January 2009 to March 2026 |
| Language restriction | English |
| Population | Adult patients with gastrointestinal malignancies |
| Search strategy | (“ultra-processed food” OR “ultraprocessed food” OR “ultra processed food” OR “NOVA classification” OR “food processing” OR “processed food” OR “Western diet” OR “Western dietary pattern”) AND (“gastrointestinal cancer” OR “digestive tract cancer” OR “colorectal cancer” OR “colon cancer” OR “rectal cancer” OR “gastric cancer” OR “stomach cancer” OR “pancreatic cancer” OR “liver cancer” OR “hepatocellular carcinoma” OR “biliary tract cancer” OR “cholangiocarcinoma” OR “esophageal cancer” OR “oesophageal cancer”) AND (“post-diagnostic” OR “postdiagnostic” OR “after diagnosis” OR “after cancer diagnosis” OR “survivorship” OR “cancer survivor” OR “cancer survivors” OR “prognosis” OR “survival” OR “mortality” OR “recurrence” OR “progression” OR “treatment response” OR “treatment tolerance” OR “quality of life”) |
| Additional search procedure | Manual screening of reference lists from eligible studies, relevant reviews, and meta-analyses was performed to identify additional studies providing direct post-diagnostic evidence, indirect survivorship evidence, or contextual epidemiological and mechanistic evidence. |
| Notes | The search was designed to map the extent, nature, and distribution of available evidence rather than to conduct a quantitative meta-analysis. Studies were classified according to level of relevance as direct post-diagnostic evidence, indirect survivorship evidence, or contextual epidemiological/mechanistic evidence. |
