# Supplementary material for: Post-diagnostic ultra-processed food exposure in gastrointestinal cancers: scoping review with narrative synthesis and clinical implications
Source: Front Nutr. 2026 Jul 14;13:1884359. doi: 10.3389/fnut.2026.1884359 (PMC13410676; doi:10.3389/fnut.2026.1884359)

**Supplementary File. Generative AI prompts and outputs used for schematic figure generation**

**Manuscript:** Post-diagnostic ultra-processed food exposure in gastrointestinal cancers: current evidence and clinical implications

**Purpose of this file:** This supplementary file reports the generative AI technology used to support the creation of schematic figures, the author-provided input instructions, the outputs received, and the author review/modification process.

**Generative AI technology:** ChatGPT, OpenAI; OpenAI image generation tool / DALL-E-based image generation. Version: ChatGPT web interface; the exact backend version of the image-generation model was not displayed in the user interface at the time of use. Provider/source: OpenAI, https://openai.com.

**Scope of use:** Generative AI was used only to support the creation of schematic figures/graphical representations. It was not used for data analysis, literature selection, interpretation of results, or formulation of the scientific conclusions. All outputs were critically reviewed and revised by the authors for scientific accuracy and consistency with the manuscript.

| **Figure** | **AI technology** | **Input prompt / author instructions** | **AI-generated output received** | **Author review and modifications** |
| --- | --- | --- | --- | --- |
| Figure 2 | ChatGPT, OpenAI; OpenAI image generation tool / DALL-E-based image generation. Exact backend version not displayed in the user interface. | Create a clean scientific schematic figure on a white background illustrating the proposed biological mechanisms linking ultra-processed food exposure with gastrointestinal cancer progression and survivorship outcomes. Include UPFs as the exposure and show major pathways including metabolic dysfunction, chronic low-grade inflammation, gut microbiota alterations, intestinal barrier dysfunction, immune modulation, food additives/processing-related compounds, and downstream clinical implications. | Schematic figure showing UPF exposure connected to biological mechanisms and potential survivorship/clinical implications. | The output was reviewed by the authors and used as a schematic illustration only. Text, labels, abbreviations, and scientific content were checked against the manuscript and revised where necessary. The figure was not used to generate scientific conclusions. |
| Supplementary Figure S1 | ChatGPT, OpenAI; OpenAI image generation tool / DALL-E-based image generation. Exact backend version not displayed in the user interface. | Recreate Figure S1 as a study selection flow diagram. Literature identification and selection process for studies included in the scoping review. Records were identified through PubMed/MEDLINE searching and manual reference screening. Include: PubMed/MEDLINE records (n=214), manual reference screening (n=18), total records (n=232), duplicates removed (n=31), records screened (n=201), records excluded (n=148), full-text articles assessed (n=53), full-text exclusions (n=28), studies included (n=25), and evidence categories. | Study selection flow diagram summarizing PubMed/MEDLINE search, manual reference screening, screening, full-text assessment, exclusions, final included studies, and evidence categories. | The output was reviewed by the authors and used only as a graphical representation of the study selection process. Counts, labels, and exclusion categories were checked against the Methods section and revised where necessary. |

**Note:** The prompts above reproduce the author-provided instructions used to generate the final schematic figures. The generated images were subsequently reviewed and, where necessary, edited by the authors before inclusion in the manuscript or supplementary material.

**AI-generated outputs received**

**Figure 2. Proposed biological mechanisms linking ultra-processed food exposure with gastrointestinal cancer progression and survivorship outcomes.**


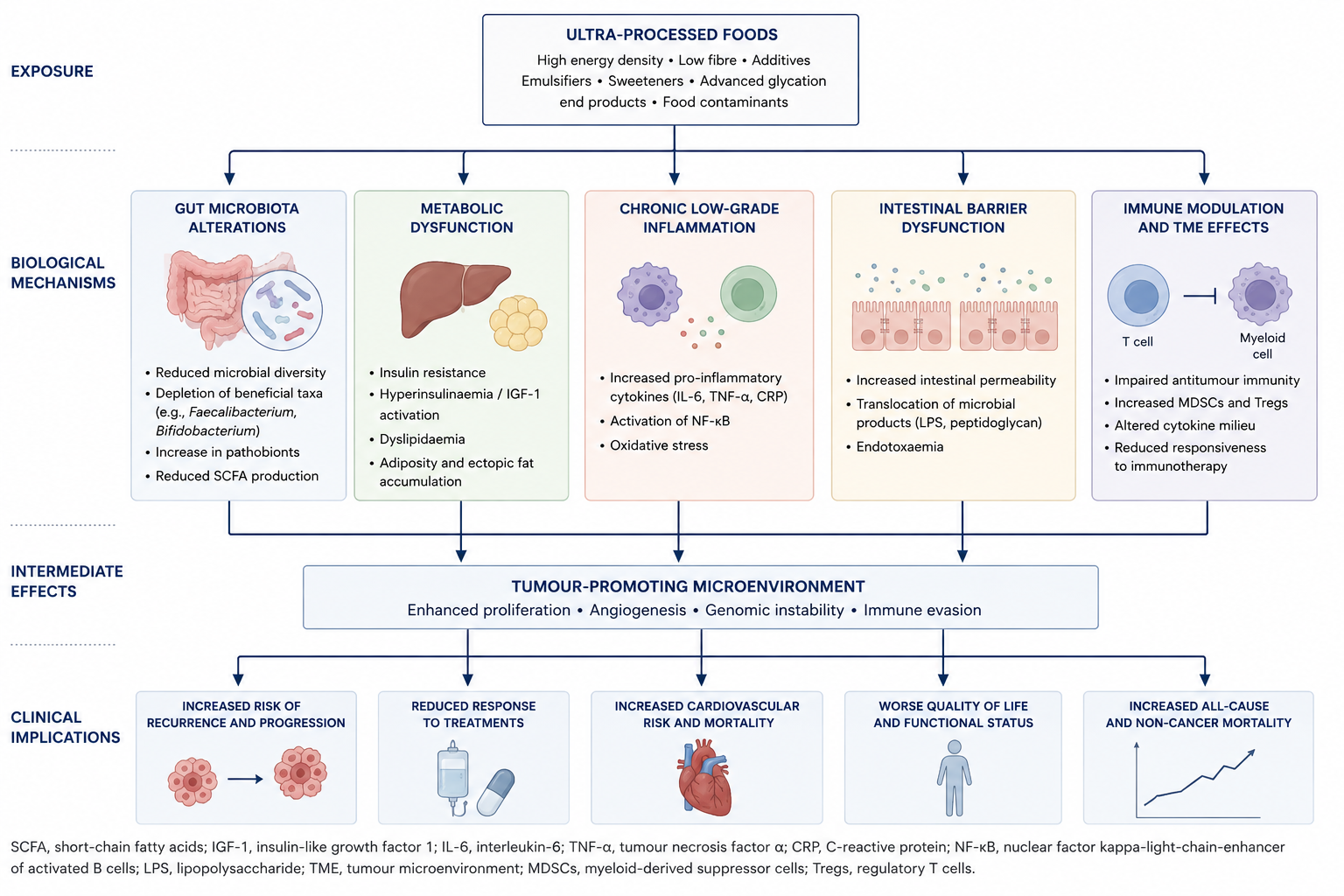


**Supplementary Figure S1. Study selection flow diagram.**


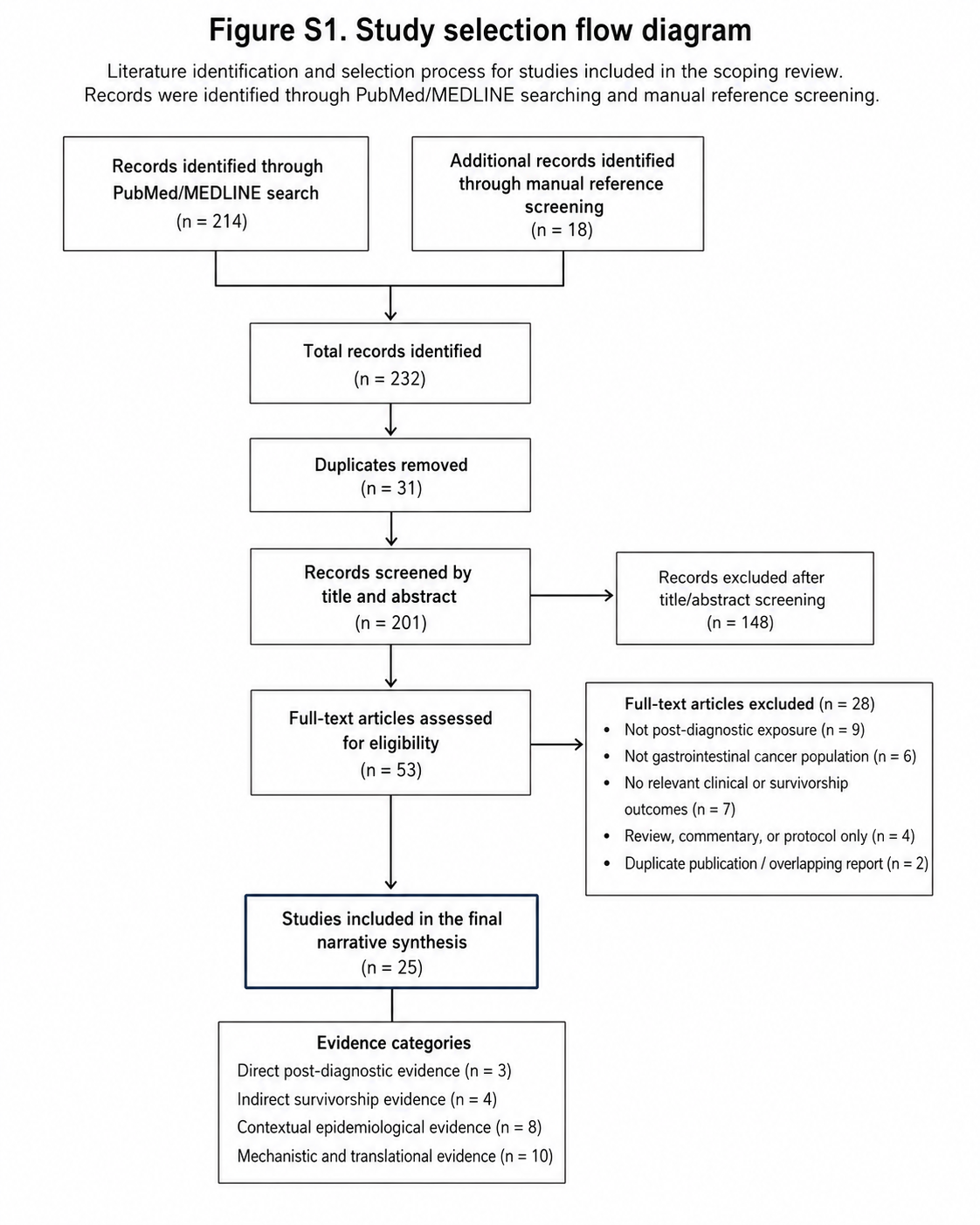

Supplement: Supplementary file 3 [file Supplementary_file_1.docx]
